# Inhibitors of Testosterone Biosynthetic and Metabolic Activation Enzymes 

**DOI:** 10.3390/molecules16129983

**Published:** 2011-12-02

**Authors:** Leping Ye, Zhi-Jian Su, Ren-Shan Ge

**Affiliations:** 1 The 2nd Affiliated Hospital, Wenzhou Medical College, Wenzhou, Zhejiang 325000, China; 2 Biopharmaceutical Research and Development Center, Jinan University, Guangzhou 510632, China; Email: su_zhijian@126.com (Z.-J.S.); 3 Population Council and Rockefeller University, 1230 York Avenue, New York, NY 10065, USA

**Keywords:** endocrine disruptor, steroidogenic enzymes, steroidogenic inhibitors, Leydig cells, male reproduction

## Abstract

The Leydig cells of the testis have the capacity to biosynthesize testosterone from cholesterol. Testosterone and its metabolically activated product dihydrotestosterone are critical for the development of male reproductive system and spermatogenesis. At least four steroidogenic enzymes are involved in testosterone biosynthesis: Cholesterol side chain cleavage enzyme (CYP11A1) for the conversion of cholesterol into pregnenolone within the mitochondria, 3β-hydroxysteroid dehydrogenase (HSD3B), for the conversion of pregnenolone into progesterone, 17α-hydroxylase/17,20-lyase (CYP17A1) for the conversion of progesterone into androstenedione and 17β-hydroxysteroid dehydrogenase (HSD17B3) for the formation of testosterone from androstenedione. Testosterone is also metabolically activated into more potent androgen dihydrotestosterone by two isoforms 5α-reductase 1 (SRD5A1) and 2 (SRD5A2) in Leydig cells and peripheral tissues. Many endocrine disruptors act as antiandrogens via directly inhibiting one or more enzymes for testosterone biosynthesis and metabolic activation. These chemicals include industrial materials (perfluoroalkyl compounds, phthalates, bisphenol A and benzophenone) and pesticides/biocides (methoxychlor, organotins, 1,2-dibromo-3-chloropropane and prochloraz) and plant constituents (genistein and gossypol). This paper reviews these endocrine disruptors targeting steroidogenic enzymes.

## 1. Introduction

Leydig cells reside in the interstitial compartment of the testis and are responsible for the production of testosterone (T). T is required for sexual development and testis descent during fetal period [1], the production of sperm in the seminiferous tubules [2] and the maintenance of accessory sex organs [3] and sexual behavior [4] at adulthood. There are two distinct populations of Leydig cells: Fetal and adult Leydig cells. Fetal Leydig cells originate in the fetal testis, and produce T. T is converted by 5α-reductase (SRD5A) to more potent androgen dihydrotestosterone (DHT) in some fetal reproductive tissues. T and DHT are required for the development of male reproductive tract and testis descent [1]. Adult Leydig cells develop during puberty and produce T that is required for maintaining spermatogenesis and male secondary sexual characteristics in adult life. Although Leydig cells only account for about 5% of all cell types in the testis at adulthood, T produced by them make over 95% of circulatory T. Chemicals that affect these cells dramatically affect androgen-dependent tissues.

There are reports of increasing incidence of cryptorchidism, hypospadias, testicular cancers and reduced fertility over the past 35 years [5,6]. Concerns have risen about the possible association of exposures to endocrine disruptors (EDs) with reproductive tract anomalies and poor sperm quality [7]. During the past decades, many environmental chemicals are considered to meet the criteria for classification as EDs, including compounds such as plasticizers (phthalates, bisphenol A), surfactants (perfluoroalkyl substances), pesticides (methoxychlor) and plant constituents (genistein and gossypol). Many EDs are classified as antiandrogens, which act against normal function of androgen-related tissues. Antiandrogenic chemicals suppress androgen production in Leydig cells, reduce their numbers, or bind to the androgen receptors (ARs) so as to block activation by androgens. In the present review, we focus on antiandrogenic EDs that directly interfere with T biosynthetic pathway and/or metabolic activation pathway. We’ll discuss the inhibition of EDs on human and rodent (rat and mouse) enzymes.

## 2. T Biosynthetic and Metabolic Activation Pathways

**Scheme 1:**
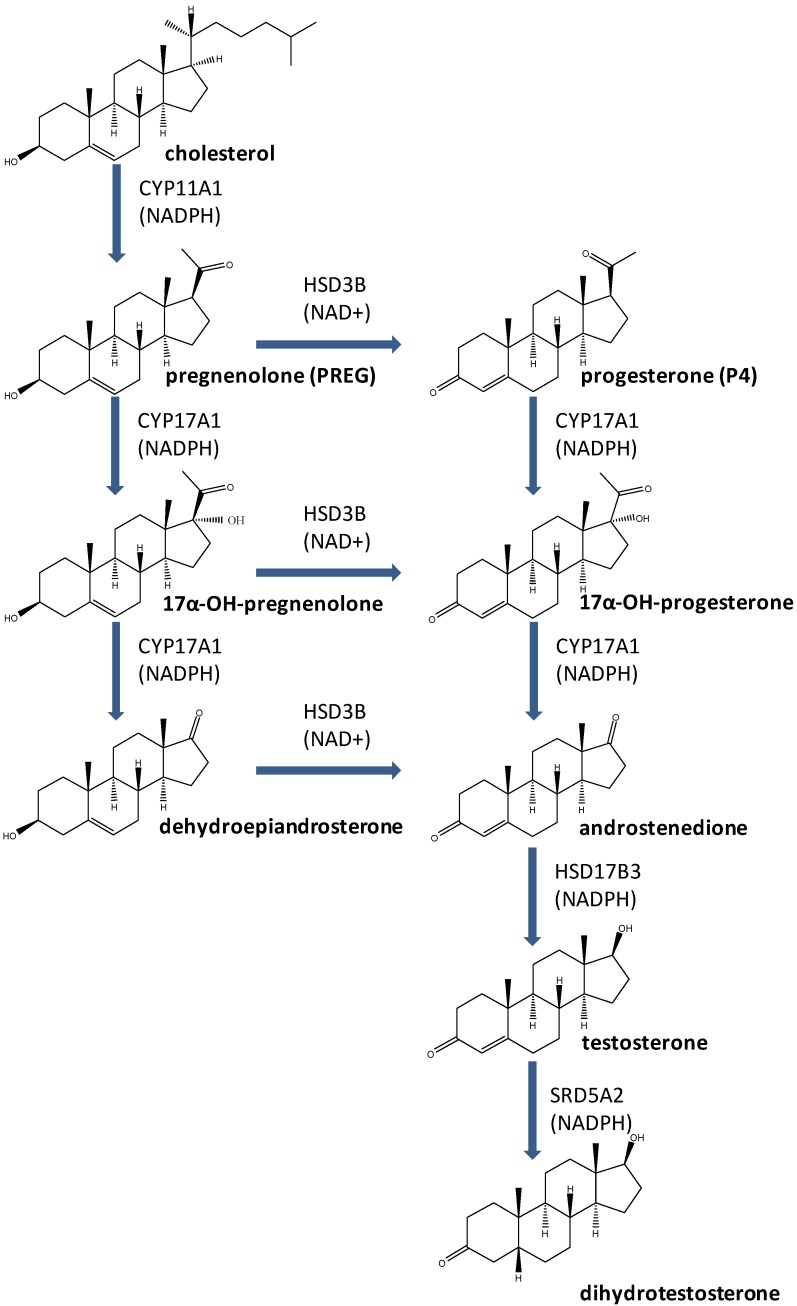
Testosterone biosynthetic and metabolic activation pathways.

In both fetal and adult Leydig cells, at least four steroidogenic enzymes are involved in T biosynthesis. T biosynthesis starts with the substrate cholesterol. The first steroidogenic enzyme is cholesterol side chain cleavage enzyme (CYP11A1) that is located in the inner membrane of the mitochondria [8]. The enzyme catalyzes three sequential reactions from cholesterol into pregnenolone. Pregnenolone diffuses from the mitochondria into the surrounding smooth endoplasmic reticulum, where other three steroidogenic enzymes are located, including 3β-hydroxysteroid dehydrogenase (HSD3B), cytochrome P450 17α-hydroxylase/17,20-lyase (CYP17A1) and 17β-hydroxysteroid dehydrogenase 3 (HSD17B3) [9]. Pregnenolone is finally converted to T by sequential reactions of these three steroidogenic enzymes. The steroid intermediates differ according to species depending upon whether the Δ^4^ or Δ^5^ pathways predominate. The Δ^4^ pathway (pregnenolone → progesterone → androstenedione → T) was first demonstrated in the rat testis [9] (Scheme 1). The Δ^5^ pathway (pregnenolone → 17α-hydroxypregnenolone → dehydroepiandrosterone → androstenedione → T) is predominant in human testis, although Δ^4^ pathway also exists [9] (Scheme 1). When T is formed, T is metabolized to more potent androgen DHT in Leydig cells or peripheral tissues by several types of 5α-reductases (SRD5A1, 2 and 3), particularly SRD5A2 that has high affinity for T [10]. DHT is very critical for male reproductive tract development in male fetus, and the mutation of *SRD5A2* gene can cause malformation of male reproductive tract [11,12]. An ED that directly inhibits one and/or more of these steroidogenic enzymes leads to lower androgen, thus as an antiandrogen.

## 3. Enzymes for T Biosynthesis and Metabolic Activation

### 3.1. CYP11A1

CYP11A1 is the first-step enzyme for the conversion of cholesterol to pregnenolone. It is encoded by human *CYP11A1* or rat *Cyp11a1*. The reaction by this enzyme occurs on the inner membrane of the mitochondria. The enzyme catalyzes three sequential reactions with each step using one molecule of cofactor NADPH. NADPH carries the energy, which is delivered by the mitochondrial electron transfer system [9]. During the catalysis, two hydroxyl groups are added to cholesterol (at C20 and C22) followed by cleavage between the added hydroxyl groups, resulting in the formation of pregnenolone [9].

### 3.2. HSD3B

HSD3B is an enzyme to catalyze the conversion of pregnenolone to progesterone in the presence of cofactor NAD^+^. It is a critical enzyme for the biosynthesis of all biologically active steroids including those in Leydig cells, adrenal, ovary and placenta [13]. HSD3B catalysis has two steps, catalyzing dehydrogenation and isomerization of a double bond in the steroid molecule, with the first dehydrogenase step being rate-limiting. There are several isoforms, with some expressed in non-classic steroidogenic tissues [9]. Two human *HSD3B* genes have been identified with 81.9% identity. Human *HSD3B1* is primarily present in placenta, while *HSD3B2* is predominantly expressed in adrenal and Leydig cells. Therefore, the review focuses on human HSD3B2 activity. In the patients with *HSD3B2* mutation [14,15], pregnenolone is not converted into progesterone in the male. In this disorder, males show varying degrees of feminization, including the development of a vagina and breast at puberty, because serum T levels are very low. Four isoforms of HSD3B in the rat have been identified [9], with each of these isoforms is the product of a distinct gene [9]. In rat Leydig cells, HSD3B1 (encoded by *Hsd3b1*) is the primary enzyme for formation of progesterone [16].

### 3.3. CYP17A1

CYP17A1 is encoded by *CYP17A1* (human) or *Cyp17a1* (rat), one enzyme with two activities. Unlike CYP11A1, which is found in the mitochondria, CYP17A1 is found in the smooth endoplasmic reticulum (SER) of Leydig cells and catalyzes two functional oxidase reactions of progesterone to 17α-hydroxyprogesterone by 17α-hydroxylase, and further 17α-hydroxyprogesterone into androstenedione by 17,20-lyase [17]. Each reaction requires cofactor NADPH [9]. The microsomal electron transfer protein cytochrome P450 oxidoreductase transfers electrons [9]. CYP17A1 catalyzes both pregnenolone and progesterone (Scheme 1). Although CYP17A1 catalyzes both hydroxylation and lysis reactions, there are species-dependent differences in the utilization of either 17α-hydroxypregnenolone (Δ5) or 17α-hydroxyprogesterone (Δ4) as substrate for the lyase reaction. The human CYP17A1 uses 17α-hydroxypregnenolone as the preferential substrate to yield dehydroepiandrosterone, whereas rat enzyme utilizes 17α-hydroxyprogesterone as the substrate to yield androstenedione. A mutation of *CYP17A1* alters the conversion of progesterone to androstenedione in the male, leading to defective masculinization that can range from partial to complete pseudohermaphroditism and breast enlargement [18,19].

### 3.4. HSD17B3

There are over fourteen 17β-hydroxysteroid dehydrogenase isoforms [16]. Only HSD17B3 is located in Leydig cells for the final conversion of androstenedione into T [20]. HSD17B3 is encoded by human *HSD17B3* or rat *Hsd17b3*. HSD17B3 requires NADPH as its cofactor. The production of T is considered an end-product. The mutation of *HSD17B3* causes various phenotypes including pseudohermaphroditism with very low circulating T in males [21,22].

### 3.5. SRD5A2

SRD5A2 is encoded by *SRD5A2* (human) and *Srd5a2* (rat). To date, three distinct SRD5As have been characterized. Human genes encode type 1 (*SRD5A1*), 2 (*SRD5A2*) and 3 (*SRD5A3*) 5α-reductases, which catalyze the conversion of T into DHT [23,24,25]. Rat genes have the similar designation (*Srd5a1*, *Srd5a2* and *Srd5a3*). The SRD5A uses NADPH as a cofactor [20,26]. A cDNA encoding human *SRD5A1* was first cloned [27]. So far, no clear mutation of SRD5A1 has been found to be associated with any human diseases. However, when human *SRD5A2* was cloned, the mutation of this enzyme was found to be associated with male pseudohermaphroditism [28]. *SRD5A2* gene is localized to human chromosome 2 [29]. Recently, human *SRD5A3* was identified after a genome wide screening of hormone-refractory prostate cancer cDNAs [30]. SRD5A3 is found not only to catalyze the formation of DHT but also to convert polyprenol to dolichol, and its mutation causes congenital glycosylation disorder, which does not affect reproduction [25]. Therefore, only SRD5A2 is associated with the development of male reproductive tract.

## 4. EDs with Direct Inhibition on Enzymes for T Biosynthesis and Metabolic Activation

Environmental chemicals can directly alter the T biosynthetic or the metabolic activation pathways. Altering one or more steps in the steroidogenesis has the potential to cause reproductive toxicity, including abnormal reproductive tract, diminished fertility and hypogonadism [31]. We list examples of chemicals that directly alter key steps in the steroidogenic pathway (Table 1). The spectrum of inhibitors has been expanded to many categories of chemicals including industrial materials (perfluoroalkyl substances, phthalates, bisphenol A), insecticides/biocides (methoxychlor and prochloraz) and plant constituents (isoflavone and gossypol). Although some other toxicants (like PCB congeners) that also interfere with steroidogenic machinery, they are not included in this review because none were reported to directly inhibit steroidogenic enzymes.

**Table 1:** Inhibitors of enzymes for testosterone biosynthesis and metabolic activation.

Enzyme	Chemicals	Use	Mode of inhibition
**Enzymes for Testosterone Biosynthesis**			
**CYP11A1**	Methoxychlor & HPTE	Insecticide	Non-competitive
	Gossypol	Plant constituent	Mixed type
	Lindane	Insecticide	Unknown
**HSD3B**	Perfluorooctane sulfonate	Surfactant	Competitive
	Perfluorooctane acid	Surfactant	Competitive
	Phthalates	Plasticizers	Competitive
	Bisphenol A	Plasticizer	Competitive
	Methoxychlor & HPTE	Insecticide	Non-competitive
	Triphenyltin	Biocide	Unknown
	Tributyltin	Biocide	Unknown
	Genistein	Plant constituent	Competitive
	Gossypol	Plant constituent	Competitive
**CYP17A1**	Bisphenol A	Plasticizer	Competitive
	Triphenyltin	Biocide	Unknown
	Tributyltin	Biocide	Unknown
	1,2-Dibromo-3-chloropropane	Insecticide	Unknown
	Prochloraz	Biocide	Unknown
	Gossypol	Plant constituent	Unknown
**HSD17B3**	Perfluorooctane sulfonate	Surfactant	Non-competitive
	Perfluorooctane acid	Surfactant	Non-competitive
	Phthalates	Plasticizers	Unknown
	Bisphenol A	Plasticizer	Competitive
	Benzophenones	UV blocker	Unknown
	Methoxychlor & HPTE	Insecticide	Non-competitive
	Triphenyltin	Biocide	Unknown
	Tributyltin	Biocide	Unknown
	Gossypol	Plant constituent	Competitive
**Enzyme for Testosterone Metabolic Activation**			
**SRD5A2**	Triphenyltin	Biocide	Non-competitive
	Tributyltin	Biocide	Non-competitive
	Genistein	Food constituent	Unknown
	Gossypol	Plant constituent	Unknown

HPTE: 2,2-bis(p-hydroxyphenyl)-1,1,1-trichloroethane.

### 4.1. Industrial Materials

#### 4.1.1. Perfluoroalkyl Substance (PFASs)

PFASs are polyfluoroalkyl compounds that are widely used for industrial and consumer products because of their unique properties of extreme stability and surface activity [32]. These chemicals are used as surfactants, adhesives and insecticides such as coatings of textiles, paper and upholstery and as reaction additives in various processes [33,34,35]. These chemicals are persistent in the environment because they are not broken down chemically and have become widespread in the environment and accumulated in wildlife and humans. Some PFASs, including perfluorooctane sulfonate (PFOS, 8 carbons + 1 sulfur), perfluorooctane acid (PFOA, 8 carbons) and perfluorohexane sulfonate (PFHxS, 6 carbons + 1 sulfur) have been classified as persistent organic pollutants in the general population rates are over 4 years in humans [36]. The levels of PFOS, PFOA and PFHxS in the blood of human subjects are related to the exposure level and duration. The serum levels of PFOS, PFOA and PFHxS in the United States in 2006 are about 14.7, 3.4 and 1.5 ng/mL, respectively [37]. A short carbon chain perfluorobutane sulfonate (PFBS, 4 carbons + 1 sulfur) has been introduced recently to replace PFOA, PFOS and PFHxS compounds. The serum elimination of PFBS is expected to be more rapid than that of PFOA or PFOS [38], thus becoming less accumulation in human bodies.

There is growing evidence that PFASs may act as EDs, interfering with the reproductive system in males. Workers in 3M in Cottage Groove of the United States that produced PFOA had higher serum level of PFOA and decreased serum T concentrations [39,40]. Laboratory animal studies also showed that rats exposed to PFOA and related chemicals had lower T levels [41,42]. One of the mechanisms by PFASs may be caused by their direct inhibition on some T biosynthetic enzymes. Apparently, PFOS and PFOA directly inhibit rat Leydig cell HSD3B. Structure activity response analysis of the inhibitory actions on rat testicular HSD3B by PFASs showed that PFASs had clear structure activity response depending on the length of carbon plus sulfur chain, with inhibitory potency of PFOS (IC_50_ = 1.3 μM) > PFOA (IC_50_ = 53.2 μM) > PFHxS (no inhibition at 250 μM) = PFBS (no inhibition at 250 μM) [32]. The mode of the inhibition on rat HSD3B is competitive against substrate pregnenolone [32]. Surprisingly, PFASs have almost no inhibitory effects on human testicular HSD3B activity [32]. In the contrast, PFOS is a very potent human testicular HSD17B3 inhibitor. The potencies are PFOS (IC_50_ = 6.0 μM) > PFOA (IC_50_ = 127.6 μM) > PFHxS (no inhibition at 250 μM) = PFBS (no inhibition at 250 μM) [32]. PFOS shows a non-competitive inhibition of human HSD17B3 [32]. Of these PFASs, only PFOA potently inhibits rat Leydig cell HSD17B3 with IC_50_ value of 17 μM [43]. The inhibition of HSD3B and HSD17B3 activities in rat Leydig cells clearly leads to the decrease of T production in Leydig cells [43]. No reports have been shown concerning the effects of PFASs on CYP11A1, CYP17A1 and SRD5A2 activities.

#### 4.1.2. Phthalates

Phthalates are synthetic compounds, which are widely used as plasticizers and solvents in a variety of polyvinyl chloride consumer products [44]. Phthalates are not chemically bound to polyvinyl chloride and easily leached out. The leached phthalates in the environment are significant because phthalates usually make up to 40% of the volume of the plastics [45]. Worldwide, manufacturers produce an estimated one billion pounds of phthalates per year [46]. Dozens of phthalates are manufactured and their difference depends on length carbon chain in the alcohol moiety. For example, dimethyl phthalate (DMP) has one carbon, and di-*n*-butyl phthalates (DBP) has four carbons in the alcohol moiety. The most abundant are diethylhexyl phthalate (DEHP) and DBP [47]. When absorbed into human body, phthalate diesters are rapidly converted into monoester metabolites [48]. Some monoester metabolites are believed to be more potent than their parent compounds for their toxicity. For example, the monoethylhexyl phthalate (MEHP), the metabolite of DEHP, has been found to be 10 times more potent than DEHP for its toxicity [49].

Phthalates have been classified as antiandrogens. Apparently, phthalates act not via blocking the androgen receptor, since they do not bind to androgen receptor [47]. *In vivo* studies using animal models have shown that DEHP and DBP indeed are antiandrogens, causing various androgen-deficient reproductive malformations, including hypospadia and indecent testis after birth, when male fetus are exposed to phthalates during gestation [50,51,52,53,54,55]. Epidemiological studies also claim that exposure to phthalates may be linked to abnormal reproductive development in human male embryos [56,57,58]. Although many mechanisms account for the reduction of T after *in vivo* or *in vitro* exposure to phthalates [47,52,53,56,57], the direct inhibitory effects of phthalates on some T biosynthetic enzymes may also be involved. It was found that the treatment of dipentyl phthalate can cause a significant decrease of CYP17A1 activity [59]. We also demonstrate that dipropyl phthalate, DBP, dipentyl phthalate, dicyclohexyl phthalate, benzyloctyl phthalate and butylbenzyl phthalate significantly inhibit both human and rat testicular HSD3B and HSD17B3 activities at concentrations of 100 μM (unpublished data). *In vitro*, the DBP metabolite monobutyl phthalate does not appear to inhibit 22-OH-cholesterol-induced T production in the fetal rat testis, indicating monobutyl phthalate does not inhibit CYP11A1 activity [60]. DEHP is not the SRD5A inhibitor either [61].

#### 4.1.3. Bisphenol A (BPA)

BPA is a synthetic compound that is used primarily in the manufacture of polycarbonate plastic and epoxy resins, and as a non-polymer additive to other plastics. Sources of human exposure to BPA include indoor air, dust ingestion and contamination of foods [62]. Various studies have demonstrated significant exposure to humans with 95% of detection in human urine samples [63,64,65]. There is clear sex differences regarding to serum BPA levels, which are significantly higher in normal men (1.49 ng/mL) compared to those of women (0.64 ng/mL). This gender difference in serum BPA levels are possibly due to difference in the androgen-related metabolism of BPA [66].

Many studies propose BPA as an estrogenic compound because it weakly binds to estrogen receptor [67,68]. BPA is also an antiandrogen, as it binds to human androgen receptor and blocks DHT-induced androgen receptor transcription activity [67,68]. Its antiandrogenic potency is comparable to the androgen receptor antagonist flutamide [67,68]. Both *in vivo* and *in vitro* exposures to BPA in rodents caused significant decreases of T production [69,70]. The inhibition of T production in rat Leydig cells has been shown to be associated with its direct inhibition on T biosynthetic enzyme activities. Although BPA has no direct inhibitory effects on CYP11A1 activity [71], it inhibits other three T biosynthetic enzymes by various degrees [72]. BPA inhibits human and rat testicular HSD3B with IC_50_s of 7.9 and 26.5 μM, and human and rat CYP17A1 activities with IC_50_s of 18.9 and 64.6 μM, respectively. BPA is also a weak human and rat HSD17B3 inhibitor with IC_50_s about 100 μM [72]. BPA is a competitive inhibitor for both HSD3B [72] and CYP17A1 [72,73] against each steroid substrate, possibly because it has very similar chemical structure to steroid substrates.

#### 4.1.4. Benzophenone (BP)

Benzophenones are the synthesized chemicals that block UV and are widely used in inks, imaging, and clear coatings in the printing industry. BPs are exposed because they migrate into food from packing [74]. Many BPs may have antiandrogenic activities. Of nine BPs (1**–**8 and 12) tested, BP-1 is the most potent inhibitor of human HSD17B3 activities with IC_50_ of 1 μM, while others have IC_50_s around 47**–**111 μM [75]. Apparently, the inhibition of BP-1 on human HSD17B3 activity is selective, since it inhibits HSD17B1 and HSD17B2 activities with IC_50_ over 20 μM and has no inhibition on HSD17B5 activity [75]. Rodent models also show BP-1 significantly inhibits T production in mouse and rat testes [75].

### 4.2. Insecticides and Fungicides

#### 4.2.1. Methoxychlor (MXC)

The organochlorine pesticide MXC is developed as a replacement for the banned pesticide 2,2-bis(*p*-chlorophenyl)-1,1,1-trichloroethane (DDT) and is widely used. MXC is a known ED to cause the reduction of luteinizing hormone (LH)-stimulated T production in rodent Leydig cells [76,77,78]. Some effects of MXC is believed to be mediated by its bioactive metabolite, 2,2-bis*(p*-hydroxyphenyl)-1,1,1-trichloroethane (HPTE) [77]. Both MXC and HPTE have estrogenic activities via binding to estrogen receptor [79]. MXC is also an antiandrogen. The androgenic effects of MXC and its metabolite HPTE are mediated via direct inhibition of T biosynthetic enzymes. MXC and HPTE directly inhibit T production in rat Leydig cells via inhibiting CYP11A1 activities starting at 100 nM [80]. Using purified pig CYP11A1, [^14^C]MXC was found to irreversibly bind to CYP11A1 and abolish the enzyme activity [81], suggesting that MXC is non-competitive inhibitor of CYP11A1. MXC also inhibits human and rat testicular HSD3B activities, with IC_50_s of 53.2 µM (human) and 46.15 µM (rat). It seems that HPTE is more potent than MXC, because it has IC_50_s of 8.2 µM (human) and 13.8 μM (rat) for HSD3B activity. The mode of MXC and HPTE on HSD3B activity is non-competitive against the substrate pregnenolone. At the concentration as high as 100 µM, MXC does not have inhibitory effects on human and rat HSD17B3 activities, while HPTE significantly inhibits human and rat HSD17B3 activities with IC_50_s of 12.1 µM (human) and 32.0 µM (rat), suggesting that MXC is metabolically activated into HPTE to inhibit HSD17B3 activity.

#### 4.2.2. Organotins

Organotins are the organometallic compounds and have been widely used as antifouling biocides for ships and fishing nets, agricultural fungicides and rodent repellents [82]. Their widespread uses have resulted in the release of increasing amounts of organotins into the environment. Organotins have been shown to be antiandrogens. For example, tributyltin causes serious defects in testicular development and function *in vivo* [68]. Studies have shown that organotins directly inhibited many T biosynthetic and metabolizing enzymes. Organotins tributyltin and triphenyltin inhibit pig CYP17A1 activities with IC_50_s of about 117 μM [83]. Tributyltin inhibits rat CYP17A1 with IC_50_ of about 50 μM [83]. Tributyltin is a primarily competitive inhibitor of rat testicular HSD3B activity with Ki of 2.4 μM [84]. Triphenyltin and tributyltin inhibited HSD17B3 activities from pig Leydig cells with IC_50_s of 48 and 148 nM, respectively [83]. Lo *et al*. [85] investigated the *in vitro* effects of triphenyltin on human T biosynthetic and metabolizing enzymes including HSD3B2, HSD17B3 and SRD5A2 activities. The IC_50_s of inhibiting HSD3B2, HSD17B3 and SRD5A2 are 4.0, 4.2 and 0.95 μM, respectively [85]. The inhibition of SRD5A2 activity may be mediated by the interaction of triphenyltin with critical cysteine residues of the enzymes [85]. The T metabolism is also performed on effects of tributyltin chloride, which inhibits human SRD5A1 and SRD5A2 with IC_50 _of 19.9 and 10.8 µM, respectively [86]. Both isoforms are not affected by tetrabutyltin or monobutyltin indicating that at least two butyl groups bound to the positively charged Sn are required for the interaction of butyltin with the enzymes [86]. The inhibition of tributyltin on SRD5A1 is competitive while that on SRD5A2 activity is irreversible [86].

#### 4.2.3. 1,2-Dibromo-3-chloropropane (DBCP)

DBCP is a pesticide, which has been used for over 20 years to control plant worms. It was banned by the US Environmental Protection Agency in 1977, because it was shown to be antiandrogen to cause infertility in male workers [87,88,89]. DBCP-exposed males may develop oligospermia and hypogonadism, but the cause is reversible [90,91,92]. The route of exposure seems to be a critical factor for the testicular toxicity of DBCP [87,88,89,93,94,95,96,89,93]. Although, many studies concluded that Leydig cells in the testis were secondary targets for DBCP toxicity, Kelce *et al*. [97] demonstrated that DBCP also had a direct inhibitory effect on the 17α-hydroxylase activity of CYP17A1 but not the 17,20-lyase activity [97].

#### 4.2.4. Lindane

Lindane is an organochlorine insecticide. Lindane was found in the human and rat testis after exposure [98,99]. It has been shown that lindane adversely affected male reproductive function in rats after *in utero* exposure and therefore it is classified as an antiandrogen [100,101,102,103,104]. Lindane inhibited human chorionic gonadotropin-stimulated T production by rat Leydig cells [101,102], suggesting that the compound might affect testicular steroidogenesis [105]. Indeed, lindane inhibits mouse CYP11A1 activity [106].

#### 4.2.5. Prochloraz

Prochloraz is an imidazole fungicide widely used for horticulture and agriculture. The action of imidazoles (e.g., ketoconazole) used as fungicides is based on the inhibition of the cytochrome P450-dependent 14α-demethylase activity that catalyzes the conversion of lanosterol to ergosterol, an essential component of fungal cell membranes [107]. Prochloraz is classified as an antiandrogen. Maternal exposure to prochloraz caused malformation of male reproductive tracts in fetal male rats and reduced steroidogenesis in the testis [108]. Prochloraz also decreased serum T levels and delayed puberty in males during the pubertal exposure [109]. This may be contributed by the direct inhibition of prochloraz on some T biosynthetic enzymes. Indeed, prochloraz inhibited rat testicular CYP17A1 activity with Ki around 1 μM [108]. Using human adrenal H295R cells, prochloraz also concentration-dependently inhibited human CYP17A1 activity [110], and the inhibition was more selective since it did not inhibit another CYP enzyme CYP11B1, which is required for glucocorticoid biosynthesis [110].

### 4.3. Plant Active Constituents

#### 4.3.1. Isoflavone (Genistein)

Genistein, a soy isoflavone, is classified as a phytoestrogen. It is widely distributed in human and animal diet. It possesses a structure similar to estrogen 17β-estradiol and can either mimic or antagonize estrogen [111]. The highest amount of flavonoids has been found in soybeans and soy food [112]. Several studies have reported on an influence by isoflavones on Leydig cell function by decreasing T production [113]. Although the exact mechanisms of genistein on T production are not clear, the direct inhibitions of some T biosynthetic enzyme activities may account for. Genistein is a potent competitive inhibitor of human and rat testicular HSD3B activity with the IC_50_ of 0.09 μM (human) and 0.64 μM (rat) [114]. Another isoflavone equol is far less potent, and it inhibited human testicular HSD3B by 42% at 100 μM. In contrast to its potent inhibition of testicular HSD3B activity, genistein had less potent inhibition on human and rat HSD17B3, and the IC_50_s are ≥100 μM [114]. Genistein inhibited human SRD5A2 activity too, and it is a much potent inhibitor of SRD5A2 than SRD5A1 [115]. Given the increasing intake of soy-based food products and their potential effect on blood androgen level, these findings are greatly relevant to public health.

#### 4.3.2. Gossypol

Gossypol is a yellowish polyphenolic compound isolated from cotton seeds, and it was once tested as a very effective male contraceptive in China [116]. Because of its possible side effects such as hypokalemia and irreversible suppression of spermatogenesis, gossypol would not be acceptable as a male contraceptive, after evaluation by World Health Organization [117]. The exposure to gossypol could be from ingestion of cotton seed oils and materials. Food and animal agricultural industries must manage cotton-derivative product levels to avoid gossypol toxicity.

Gossypol has direct inhibition on some steroidogenic enzymes. Gossypol at 17–34 μM significantly inhibited the conversion of 25-hydroxycholesterol into pregnenolone and pregnenolone into progesterone in bovine luteal cells, suggesting that gossypol inhibits CYP11A1 and HSD3B activities [118]. Indeed, gossypol inhibited CYP11A1 from bovine adrenal mitochondria at 30 μM [119]. Gossypol is the very potent inhibitor of human and rat testicular HSD3B activities with IC_50_s of 3–5 µM for human and 0.2 µM for rat’s enzyme [120]. Gossypol potently inhibited human and rat HSD17B3 with clear enantiomer-specific differences. (−)-Gossypol inhibited human and rat HSD17B3 activities with IC_50_s of 0.36 and 3.43 μM, respectively, while the (+)-gossypol is slightly less potent and inhibited human and rat HSD17B3 activities with IC_50_ of 1.13 μM and 10.93 μM, respectively [120]. Gossypol inhibited T metabolizing enzymes SRD5A, and its inhibitory effect is more potent for SRD5A1 than SRD5A2 activity [115].

## 5. Summary and Conclusions

Leydig cells of the testis are responsible for the biosynthesis and secretion of androgens, which is critical for developmental and reproductive function in the male. Disruption of T biosynthesis and metabolic activation by EDs can cause sexual dysfunction, infertility or sterility. Many EDs were found to act directly on enzyme activity in Leydig cells. The impaired function of Leydig cells is displayed by a decrease in T production as a consequence of the suppressed CYP11A1, CYP17A1, HSD3B and HSD17B3 activities. The direct inhibition on SRD5A2 may also contribute to the abnormal development of male reproductive tract. However, our knowledge on the different EDs for disruption of particular target molecules involved in steroidogenesis is still limited, further studies are warranted to assess the effects of EDs on male fertility.
